# Isolation, Structure Elucidation and In Silico Prediction of Potential Drug-Like Flavonoids from *Onosma chitralicum* Targeted towards Functionally Important Proteins of Drug-Resistant Bad Bugs

**DOI:** 10.3390/molecules26072048

**Published:** 2021-04-02

**Authors:** Shakeel Ahmad Khan, Shafi Ullah Khan, Fozia, Najeeb Ullah, Mohibullah Shah, Riaz Ullah, Ijaz Ahmad, Amal Alotaibi

**Affiliations:** 1 Department of Chemistry, Kohat University of Science & Technology, Kohat 26000, Pakistan; shakeelchemist@gmail.com; 2 School of Pharmacy, Monash University, Bandar Sunway, Subang Jaya 47500, Malaysia; shafiullahpharmd@gmail.com; 3 Department of Pharmacy, Abasyn University, Ring Road, Peshawar 25120, Pakistan; 4 Biochemistry Department, KMU Institute of Medical Sciences, Kohat 26000, Pakistan; drfoziazeb@yahoo.com; 5 Department of Biochemistry, Bahauddin Zakariya University, Multan 60800, Pakistan; najeebkhattak@bzu.edu.pk (N.U.); mohib@bzu.edu.pk (M.S.); 6 Department of Pharmacognosy, College of Pharmacy, King Saud University Riyadh Saudi Arabia, Riyadh 11495, Saudi Arabia; 7 Basic Science Department, College of Medicine, Princess Nourah bint Abdulrahman University, Riyadh 11671, Saudi Arabia

**Keywords:** *Onosma chitralicum*, flavonoids, antimicrobial activities, SAR, beta-hydroxyacyl dehydratase

## Abstract

Admittedly, the disastrous emergence of drug resistance in prokaryotic and eukaryotic human pathogens has created an urgent need to develop novel chemotherapeutic agents. *Onosma chitralicum* is a source of traditional medicine with cooling, laxative, and anthelmintic effects. The objective of the current research was to analyze the biological potential of *Onosma chitralicum,* and to isolate and characterize the chemical constituents of the plant. The crude extracts of the plant prepared with different solvents, such as aqueous, hexane, chloroform_,_ ethyl acetate, and butanol, were subjected to antimicrobial activities. Results corroborate that crude (methanol), EtoAc, and *n*-C_6_H_14_ fractions were more active against bacterial strains. Among these fractions, the EtoAc fraction was found more potent. The EtoAc fraction was the most active against the selected microbes, which was subjected to successive column chromatography, and the resultant compounds **1** to **7** were isolated. Different techniques, such as UV, IR, and NMR, were used to characterize the structures of the isolated compounds **1–7**. All the isolated pure compounds (**1**–**7**) were tested for their antimicrobial potential. Compounds **1** (4′,8-dimethoxy-7-hydroxyisoflavone), **6** (5,3′,3-trihydroxy-7,4′-dimethoxyflavanone), and **7** (5′,7,8-trihydroxy-6,3′,4′-trimethoxyflavanone) were found to be more active against *Staphylococcus aureus* and *Salmonella Typhi*. Compound **1** inhibited *S. typhi* and *S. aureus* to 10 ± 0.21 mm and 10 ± 0.45 mm, whereas compound **6** showed inhibition to 10 ± 0.77 mm and 9 ± 0.20 mm, respectively. Compound **7** inhibited *S. aureus* to 6 ± 0.36 mm. Compounds **6** and **7** showed significant antibacterial potential, and the structure–activity relationship also justifies their binding to the bacterial enzymes, i.e., beta-hydroxyacyl dehydratase (HadAB complex) and tyrosyl-tRNA synthetase. Both bacterial enzymes are potential drug targets. Further, the isolated compounds were found to be active against the tested fungal strains. Whereas docking identified compound **7**, the best binder to the lanosterol 14α-demethylase (an essential fungal cell membrane synthesizing enzyme), reported as an antifungal fluconazole binding enzyme. Based on our isolation-linked preliminary structure-activity relationship (SAR) data, we conclude that *O. chitralicum* can be a good source of natural compounds for drug development against some potential enzyme targets.

## 1. Introduction

Microorganisms, including viruses, bacteria, and others, are potentially harmful to other living organisms, mainly humans. Bacteria largely become resistant to antibiotics, throughout the world, as examined in many hospital studies [1]. For this reason, the discovery of new and modified broad-spectrum antibiotics is very important and essential to overcome diseases. There are many sources of antimicrobials, but plants are rich sources of them. Plants are used in various countries; however, their medicinal use is central in many powerful and effective medicines [2]. Herbs are used to treat different diseases due to their efficacy and low cost, however, doctors often hesitate to recommend them because of a lack of knowledge about herbs, concerns about liability, product safety, and the existence of harmful ingredients [3]. Many bacterial strains acquired resistance against various antibiotics due to changes in their structures, mutations in their genes, and most importantly, due to excessive unselective intake of antibiotics for the treatment of various infectious diseases. All of these modifications result in the usefulness of most antibiotics and generate a renewed interest in herbal medicines [4].

The name *onosma* derived from “Osma” (Latin word) means smelly, and was used, for the first time, by Linnaeus in modern botanical classification. About 150 types of known species are included in this genus *Onosma* (Boraginaceae), and distributed in Asia, including China (39 species), Turkey (95 species), Pakistan (eight species), and others (eight species) [5]. A literature survey revealed that very little phytochemical work has been carried out on the genus *Onosma,* and only some flavonoids [6], naphthoquinones [7], alkaloids [8], and phenolic compounds [9] have so far been reported. *Onosma chitralicum* is a well-known medicinal plant belonging to the genus *Onosma*. *Onosma chitralicum* plant is mostly distributed in tropical and temperate regions, especially the Mediterranean regions. The *Onosma chitralicum* plant is a perennial herb, having multi-branched stems. Leaves and stems are densely hairy, with extensively growing hair. The flowers of this plant are nearly actinomorphic and bisexual [5].

*Onosma chitralicum* plants have different biological properties, including laxative, cooling, and anthelmintic effects among others. This plant also shows effective treatment in derangements of blood, the diseases of the eye, abdominal pain, bronchitis, wound, fever, and pile. In Pakistan, this plant is locally used by the people of Chitral to treat the diseases of the eye, as well as wounds [10].

Although *Onosma chitralicum* is famous for its medicinal properties, it has not been investigated for this purpose. The objective of this study was to evaluate the biological activities of crude extract, as well as different solvents, soluble fractions of the *Onosma chitralicum* (medicinal plant). The most active fraction was then used for the isolation of chemical constituents and biological activities of those isolated compounds were then performed.

In this article, we report the antibacterial and antifungal activities of the crude extract, solvents soluble fractions, and new source compounds (**1**–**7**) isolated for the first time from *Onosma chitralicum*, but reported already from different sources as referenced in the Materials and Methods section, which were individualized by various techniques named one-dimensional (1D) and two-dimensional (2D) NMR.

## 2. Results and Discussion

**Figure 1:**
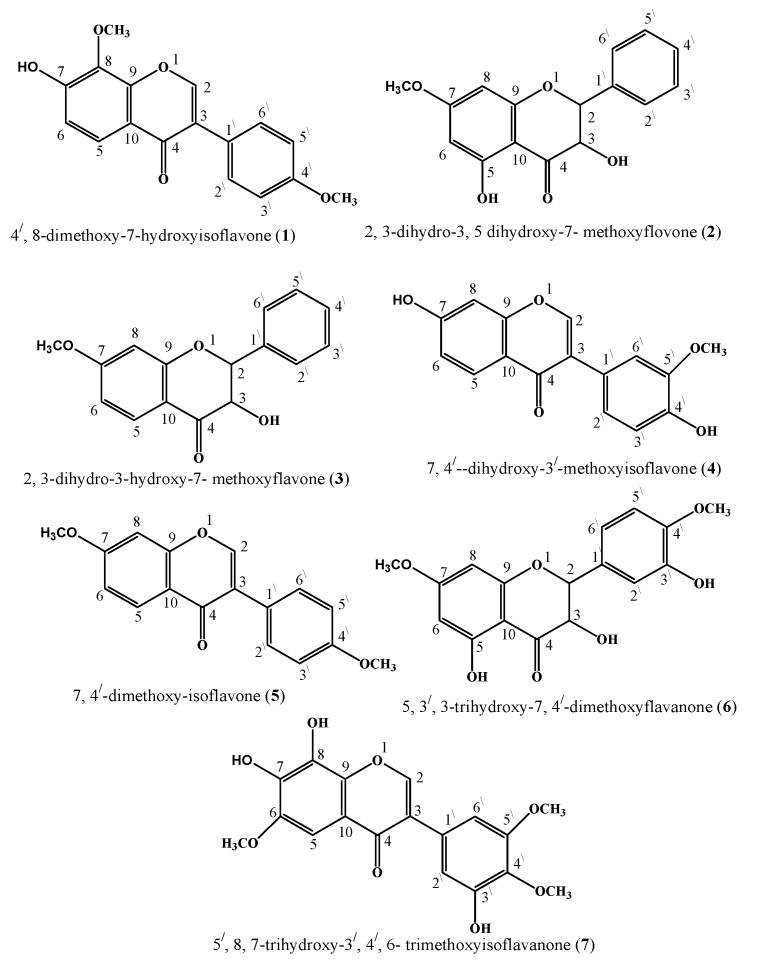
Structures of compounds (**1**–**7**).

*Onosma chitralicum* plant crude extract, fractions of different solvents and compounds **1** to **7** (shown in Figure 1), underwent antimicrobial evaluation to study their biological importance. Different solvents, either polar or non-polar (methanol, *n*-hexane, chloroform, ethyl acetate*, n*-butanol and aqueous) soluble fractions, were active against the tested microbial specimen. For antibacterial assays, five bacterial species, i.e., *Escherichia coli, Staphylococcus aureus, Pseudomonas aeruginosa, Bacillus subtilis, Salmonella typhi;* and for antifungal assays, five fungal species, i.e., *Aspergillus flavus, Aspergillus fumigates, Aspergillus niger*, *Fusarium solani, Candida glabrata* strains, were used.

### 2.1. Antibacterial Activity of Fractions

The antibacterial activities of *Onosma chitralicum* against five bacterial strains (shown in Table 1) were found active against all. The fraction of ethyl acetate (EtoAc), crude, and hexane (*n-*C_6_H_14_) fraction of *Onosma chitralicum* revealed a more potent activity than chloroform (CHCl_3_), *n*-butanol (BuOH), and aqueous fractions against various strains. The activity of ethyl acetate fraction (16.0 mm) was shown against *S. typhi,* while against *E. coli* and *S. aureus* with a 12 mm inhibition zone for each. The activity of methanol/crude fraction against *S. typhi* strain (17 mm), *E. coli* strain (13 mm), and the *S. aureus* strain (13 mm) are shown in Table 1.

### 2.2. Antifungal Activity of Fractions

The antifungal activities of *O. chitralicum* were subjected to five fungal strains and the results are given in (Table 2). In the antifungal assay, ethyl acetate and methanol/crude fractions of *O. chitralicum* were not only excellent, but also active against all fungal strains as compared to CHCl_3_, *n*-hexane, BuOH and, aqueous fractions. Methanolic fraction’s activity against *Aspergillus flavus* strain was 65 mm*, Fusarium solani* was 63 mm, and *Aspergillus niger* was 58 mm, whereas the ethyl acetate fraction’s activity against*, A. flavus* strain was 60 mm; details are elaborated in Table 2.

### 2.3. Bioscreening of Compounds

Antibacterial and antifungal activities of compounds **1** to **7** were performed, applying previously described disc and agar diffusion methods, respectively, as referenced in the Materials and Methods section, in detail. The zone of inhibition of isolated compounds **1** to **7** has been tabulated in (Table 3 and Table 4). Compounds **1**, **4**, **5,** and **6** were active against all the tested range of bacteria, while compounds **2**, **3,** and **7** were less diverse in their activity against some bacteria. In antifungal activities, all seven compounds showed activity; however, compounds **1**, **3**, **4**, **5,** and **6** were more active than compounds **2** and **7**.

### 2.4. Structure–Activity Relationship of the Reported Compounds

Flavonoids are a class of organic secondary plant phenolic compounds with significant antioxidant and chelating properties. In the human diet, they are most concentrated in fruits, vegetables, wines, teas, and cocoa. Their cardioprotective effects stem from the ability to inhibit lipid peroxidation, chelate redox–active metals, and attenuate other processes involving reactive oxygen species [11]. Despite some inconsistent lines of evidence, several structure–activity relationships are well established in vitro. Multiple hydroxyl groups confer upon the molecule substantial antioxidant, chelating, and pro-oxidant activity. Methoxy groups introduce unfavorable steric effects and increase lipophilicity. A double bond and carbonyl function in the heterocycle or polymerization of the nuclear structure increases activity by affording a more stable flavonoid radical through conjugation and electron delocalization. Further investigation of the metabolism of these phytochemicals is justified to extend structure–activity relationships (SARs) to preventive and therapeutic nutritional strategies [11].

The effectiveness of flavonoids is reflected in the double bond between C-2 and C-3, which is responsible for the planar structure, as well as for conjugation. Both these characteristics are involved in the antioxidant activity of flavonoids [12]. In the isolated compounds, **1**, **4**, **5,** and **7** reflect this property. Another important combination of functional groups and their position is the hydroxyl group at positions 5 and 3 along with the oxo group at position 4. This combination also contributes to the antioxidant activity of flavonoids [12]. Here, in our reported structures, compounds **2** and **6** have OH at C-3, 4-oxo group, and C-5-OH group. A 2,3-double bond in ring-C further increases the antioxidant effects of ring B hydroxyl groups by extending electron delocalization [13]. A double bond conjugates with 4-oxo function, which increases electron delocalization to ring B. This double bond conjugation and 4-oxo functionality are present in active compounds **1** and **7,** with compound **7,** the functional OH group at C-5’. The phenoxy radicals produced here are stabilized by the resonance effect of the aromatic nucleus [14].

The hydroxyl substitution pattern is an indicator of antibacterial activity for flavones, and the additional hydroxyl group at the 4’ position significantly increases the activity, while the methylation of these hydroxyl groups reduces the antibacterial potential at different levels. Moreover, the addition of the 3-hydroxyl group seems to enhance the activity, indicating flavonols may be better antibacterial agents than flavones [15,16]. Here, in our reported compounds, **1**, **5**, **6**, **7** have methoxy substitution at position 4’, whereas compound **4** has hydroxyl group at this position.

Based on structure–activity relationships of flavonoids, the double bond between carbon 2’ and 3’ in the B-ring, OH groups at C-7 in the ring-A collectively played an important role in antimicrobial activities [17]. In our study, the first condition of the double bond between carbon 2’ and 3’ in ring-B is present in all compounds **1**–**7**, the second condition of OH group at C-7 in ring-A is exhibited by compounds **1**, **4**, **7**.

**Figure 2:**
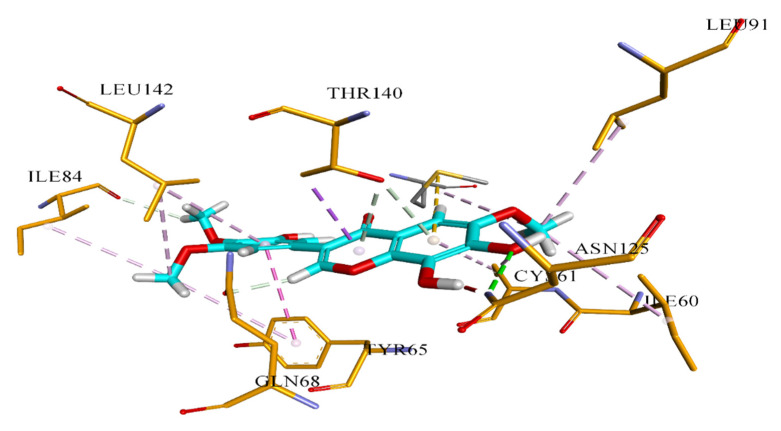
Putative binding interaction of compound **7** against bacterial beta-hydroxyacyl-ACP dehydratase HadAB.

**Figure 3:**
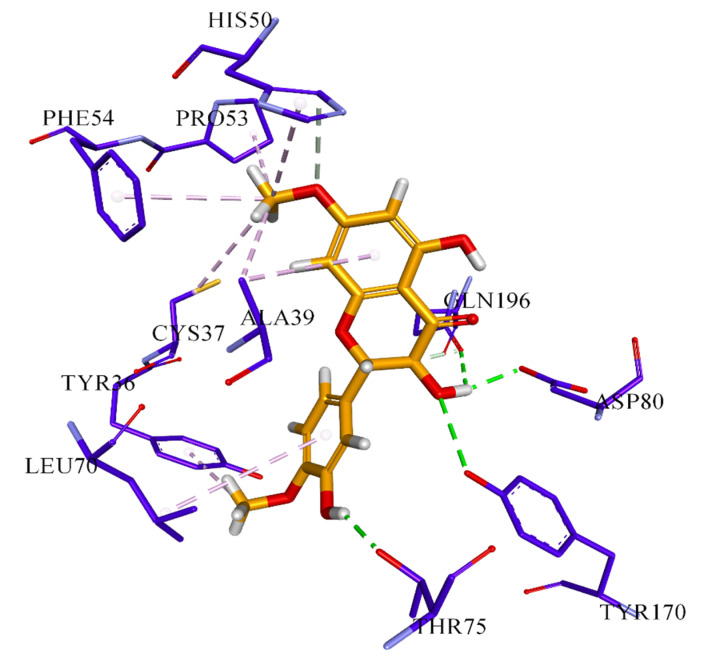
Putative binding interaction of compound **6** against bacterial *S. aureus* tyrosyl-tRNA synthetase.

**Figure 4:**
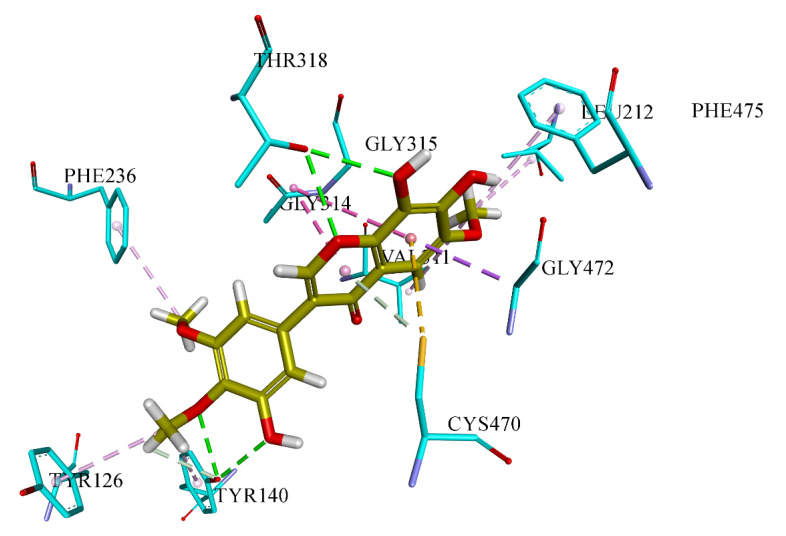
Putative binding interaction of compound **7** against lanosterol 14α-demethylase enzyme.

Further, it is reported that the hydroxyl group at positions 3, 4, and 5 in ring-B of flavonoids is responsible for antimicrobial activities against *P. vulgaris* and *S. aureus* [18]. In our results, compound **4** has OH group at C-4’ and is active against *E. coli*, compound **6** at C-3’ shows significant inhibition activity against *S. aureus* and *S. typhi,* and compound **7** at C-5’ is active against *S. aureus*. Moreover, these isolated compounds **4**, **6,** and **7,** show the highest binding affinity to the bacterial and fungal proteins in docking results. Detailed binding interaction of compound **7** against beta-hydroxyacyl-ACP dehydratase HadAB is shown in Figure 2. Compound **6,** showing strong binding affinity against the bacterial *S. aureus* tyrosyl-tRNA synthetase target, is shown in Figure 3. Whereas, interaction of compound 7 with *S. cerevisiae* lanosterol 14α-demethylase (CYP51) is provided in Figure 4. These results are summarized in Table 5, Table 6, Table 7, Table 8, Table 9, Table 10 and Table 11.

Interestingly, a report published by Stapleton and colleagues explains that substitution with C-8 and C-10 chains also enhanced the anti-*staphylococcal* activity of flavonoids [19], as compounds **1** and **7** have this substitution with OCH3 and OH groups, respectively. It was also reported by Osawa and colleagues that 5-hydroxyflavones and 5-hydroxyisoflavones with additional hydroxyl groups at the 7 and 4′ positions lost the inhibitory activity [20], whereas, in compound **6** (active) the positions 7 and 4′ are substituted with OCH3 and can be the reason for its activity. Besides the unsaturation and hydroxylation in-ring system, another most important function in the structure of flavonoids that relates its antifungal activity is methylation. The main classes of antimicrobial and antiviral flavonoids found in medicinal plants are methylated flavones and flavonols. It is reported that methylated flavonoids have antimicrobial activities against bacteria *S. aureus* and fungus *C. albicans* [18]. In our case, all seven purified compounds are methylated. However, the antimicrobial activity of each compound is different.

As demonstrated in Table 3 and Table 4, compound **1** has promising antimicrobial activity against the selected bacterial and fungal strains due to the best position of functional groups, i.e., hydroxylation at position 7 of ring A, presence of double bond between carbon 2’ and 3’ in-ring B, and delocalization of ring-C with ring B. The next active compound is **6** with active OH at C-3’, with a double bond between C-2 and C-3, showing good inhibition activity, and it has OH at C-3 with 4-oxo group and C-5–OH group in conjugation, stabilizing, and conjugating the ring system. The compounds **2**, **3,** and **6** have carbon 2 and 3 saturated, which makes them non-planar and more reactive in comparison.

### 2.5. Molecular Docking Studies

Nowadays molecular docking calculations are broadly used for investigating the binding affinities of ligands with target structure. Molecular docking studies were performed for all seven isolated and characterized compounds, to investigate the putative binding orientation within the active site of three bacterial proteins and one fungal target protein. Bacterial *Staphylococcus aureus* tyrosyl-tRNA synthetase and topoisomerase-II DNA gyrase, as well as *Mycobacterium tuberculosis* beta-hydroxyacyl-ACP dehydratase HadAB, whereas the Fungal *Saccharomyces cerevisiae* lanosterol 14α-demethylase (CYP51), are attractive therapeutic targets for the design and development of antibacterial and antifungal drugs. The crystal structure coordinates of the bacterial FabZ complexed with inhibitor Fisetin were retrieved from the Protein Data Bank PDB (PDB code: 4RLT) [21], *Staphylococcus aureus* tyrosyl-tRNA synthetase in with complex with SB-239629 (PDB code: 1JIJ) [22], topoisomerase-II DNA gyrase in complex with ciprofloxacin and DNA (PDB id: 2XCT) [23], and *Saccharomyces cerevisiae* lanosterol 14α-demethylase (CYP51) in complex with fluconazole at the active site (PDB code: 4WMZ) [24].

The binding affinity of all seven compounds against the aforementioned four target proteins was evaluated based on Chemguass4 score implemented in FRD docking software. Fast rigid exhaustive docking (FRED) uses a multi-conformer docking procedure, which separately creates a set of low-energy conformers, and then does rigid docking for each conformer. Three parameters were used for docking analysis: the binding affinity expressed in kcal/mol, the interactions between the ligand atoms, amino acid residues of the target protein, and the distance of these interactions. The binding affinity of these compounds shows the highest binding value with *M. tuberculosis* beta-hydroxyacyl-ACP dehydratase HadAB compared to the lowest from the *S. aureus* topoisomerase II DNA gyrase, as shown in Table 5, Table 7, Table 9 and Table 11. Compound **7** was found to form the most stable complex with beta-hydroxyacyl-ACP dehydratase HadAB and also *S. cerevisiae* lanosterol 14α-demethylase (CYP51), owing to the Chemguass4 score of −15.2557, −11.57**,** respectively. Whereas, compound **6** indicated strong binding interaction with *S. aureus* tyrosyl-tRNA synthetase at a Chemguass4 score of −13.12. Moreover, the detailed binding interaction of compound **6,** showing strong binding affinity against the bacterial *S. aureus* tyrosyl-tRNA synthetase target, is shown in Figure 3 and summarized in Table 8.

The bacterial fatty synthase-II is lacking in mammals (mammal have fatty acid synthase-I) and, therefore, it has recently attracted much attention as a potential antibacterial drug target. FabZ is a ubiquitous component of the bacterial fatty acid synthesis pathway. This enzyme catalyzes the dehydration of beta-hydroxyacyl-ACP to trans-2-acyl-AC and, therefore, called Had (Hydroxyacyl dehydratase) [25]. FabZ is a ubiquitous component of the bacterial fatty acid synthesis pathway, ubiquitously present in Gram-positive and Gram-negative bacteria [25]. This enzyme has been extensively studied as an effective drug target in various pathogenic bacteria, including *S. aeruginosa* [26], *Helicobacter pylori* [27], etc. Flavonoids, such as fisetin, have been shown to inhibit the FabZ in a number of studies, by perturbing the binding of the substrate in the active site and obstruct the placement of fatty acids in the channel in the HadA at the dimer interface [25]. Fisetin is a plant-derived polyphenol flavonoid, having antibacterial, antioxidant, and anticancer activities [28].

**Table 5:** FRED score of all compound (compounds 1–7) against docking in beta-hydroxyacyl-ACP dehydratase HadAB (Protein Data Bank (PDB) ID: 4rlt) as a potential antibacterial target.

Compound Code	FRED Chemguass4 Score
Compd_1	−12.3517
Compd_2	−13.9049
Compd_3	−13.2884
Compd_4	−14.5556
Compd_5	−12.9227
Compd_6	−15.1099
Compd_7	−15.2557
4RLT_cc	−14.8466

**Table 6:** Detailed analysis of compound **7** against docking in beta-hydroxyacyl-ACP dehydratase HadAB (PDB ID: 4rlt) as a potential antibacterial target.

Type of Bond	Distance	Interacting Amino Acid of Target
Hydrogen Bond	3.04121	A:ASN125:ND2
Hydrogen Bond	2.21139	A:GLN68:OE1
Hydrogen Bond	2.76714	A:ILE84:O
Hydrogen Bond	3.41761	A:THR140:OG1
Hydrogen Bond	3.87218	A:THR140:OG1
Pi-Sigma	3.87034	A:THR140:CG2
Pi-Sulfur	5.56442	B:MET60:SD
Pi-Pi T-shaped	4.88136	A:TYR65
Hydrophobic	4.79327	A:ILE84
Hydrophobic	4.89475	A:LEU142
Hydrophobic	5.17456	A:ILE60
Hydrophobic	3.9138	A:CYS61
Hydrophobic	4.94236	A:LEU91
Hydrophobic	5.33226	B:MET60
Hydrophobic	5.37435	A:LEU142
Hydrophobic	5.37548	A:CYS61
Hydrophobic	5.48466	A:TYR65

**Table 7:** FRED score of all compound (compounds **1**–**7**) against docking in *S. aureus* tyrosyl-tRNA synthetase (PDB ID: 1jij) as a potential antibacterial target.

Compound Code	FRED **Chemguass4** Score
Compd_1	−11.39
Compd_2	−10.67
Compd_3	−11.30
Compd_4	−11.75
Compd_5	−9.79
Compd_6	−13.12
Compd_7	−12.93
Co-crystalized ligand	−14.64

Lanosterol 14α-demethylase enzyme catalyzes an essential step in the synthesis of ergosterol, which is an essential component of the fungal cell membrane. CYP51 is the target of azoles, the most popular class of antifungal drugs. There has been a considerable amount of research interest into this enzyme and the azoles because of the dramatically increasing number of drug resistance among certain fungal species. Molecular docking of all seven compounds against Lanosterol 14α-demethylase enzyme revealed that compound **7** was forming the most stable complex with the lanosterol 14α-demethylase enzyme, owing to the Chemguass4 score of −11.57. Detailed binging interactions of compound **7** are shown in Figure 4 and summarized in Table 10.

**Table 8:** Detailed analysis of compound **6** against docking in *S. aureus* tyrosyl-tRNA synthetase (PDB ID: 1jij) as a potential antibacterial target.

Type of Bond	Distance (Å)	Interacting Amino Acid of Target
Hydrogen Bond	3.02926	A:TYR170:OH
Hydrogen Bond	2.11983	A:THR75:OG1
Hydrogen Bond	2.07234	A:ASP80:OD2
Hydrogen Bond	2.66497	A:GLN196:OE1
Hydrogen Bond	3.53206	A:HIS50:CD2
Hydrogen Bond	2.08403	A:GLN196:OE1
Hydrophobic	4.41402	A:ALA39
Hydrophobic	5.21473	A:CYS37
Hydrophobic	4.58554	A:PRO53
Hydrophobic	4.46255	A:TYR36
Hydrophobic	4.44049	A:HIS50
Hydrophobic	4.45421	A:PHE54
Hydrophobic	5.31681	A:LEU70
Hydrophobic	4.82878	A:ALA39

**Table 9:** FRED score of all compounds (compound **1**–**7**) against docking in lanosterol 14α-demethylase enzyme (PDB ID: 4wmz) as a potential antifungal target.

Compound Code	FRED Chemguass4 Score
Compd_1	−10.03
Compd_2	−10.61
Compd_3	−9.88
Compd_4	−11.07
Compd_5	−10.06
Compd_6	−11.44
Compd_7	−11.57
Co-crystalized ligand	−11.25

**Figure 5:**
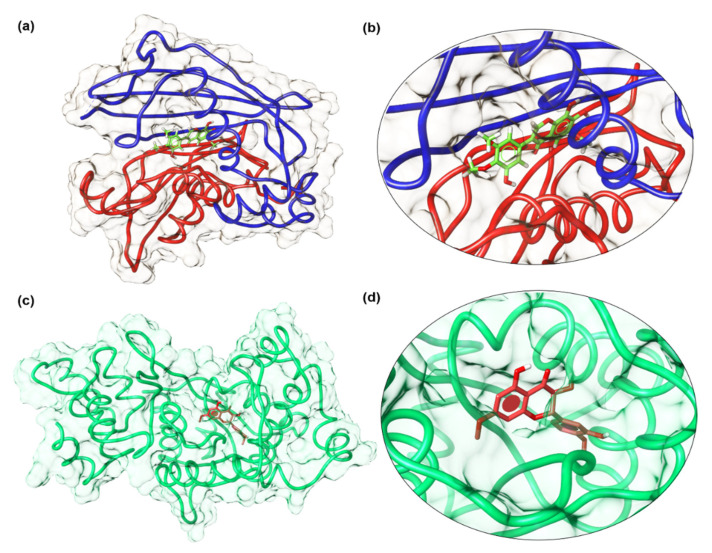
Binding pocket visualization of important bacterial potential enzyme targets docked with isolated compounds from *Onosma chitralicum*. (**a**,**b**) Compound **7** docked with *M. tuberculosis* beta-hydroxyacyl-ACP dehydratase HadAB, and bound in the same location, i.e., the channel at the dimer interface where the co-crystallized ligand fisetin was found in the pocket in the reported PDB. Compound **7** is shown in green stick representation and the dimeric protein with blue and green protomers. (**c**,**d**) Docked compound **6** (shown in red sticks) binds to the active site pocket of *S. aureus* tyrosyl-tRNA synthetase (in green ribbon).

The docking process was validated by redocking the co-crystallized ligand and found bound to the binding pocket with similar ligand–protein interactions. Binding pockets of Mycobacterium tuberculosis beta-hydroxyacyl-ACP dehydratase HadAB (PDB code: 4RLT) with docked compound **7** (5′,7,8-trihydroxy-6,3′,4′-trimethoxyflavanone), and S. aureus tyrosyl-tRNA synthetase (PDB ID: 1JIJ) with docked compound 6 (5,3′,3-trihydroxy-7,4′-dimethoxyflavanone) are visualized by molecular visualization program UCSF chimera [29], as shown in Figure 5.

**Table 10:** Detailed analysis of compound **7** against docking in lanosterol 14α-demethylase enzyme (PDB ID: 4wmz) as a potential antifungal target.

Type of Bond	Distance (Å)	Interacting Amino Acid of Target
Hydrogen Bond	3.11333	A:TYR140:OH
Hydrogen Bond	2.85993	A:TYR140:OH
Hydrogen Bond	2.96137	A:THR318:OG1
Hydrogen Bond	2.96662	A:THR318:OG1
Hydrogen Bond	3.03261	A:TYR140:OH
Pi-Donor Hydrogen Bond; Pi-Sulfur	3.98031	A:CYS470:SG
Pi-Sigma	3.72732	A:GLY472:CA
Pi-Sulfur	4.25042	A:CYS470:SG
Amide-Pi Stacked	4.6864	A:GLY314:C,O;GLY315:N
Amide-Pi Stacked	3.99431	A:GLY314:C,O;GLY315:N
Hydrophobic	4.82112	A:LEU212
Hydrophobic	5.10465	A:VAL311
Hydrophobic	4.45331	A:TYR126
Hydrophobic	5.29002	A:TYR140
Hydrophobic	4.8273	A:PHE236
Hydrophobic	4.52325	A:PHE475

**Table 11:** FRED score of all compound (compounds **1**–**7**) against docking in topoisomerase II DNA gyrase (PDB ID: 2xct) as a potential antibacterial target.

Compound Code	FRED **Chemguass4** Score
Compd_1	−2.20
Compd_2	−2.43
Compd_3	−2.47
Compd_4	−3.35
Compd_5	−1.70
Compd_6	−2.29
Compd_7	−2.17
Co-crystalized ligand	−3.25

**Figure 6:**
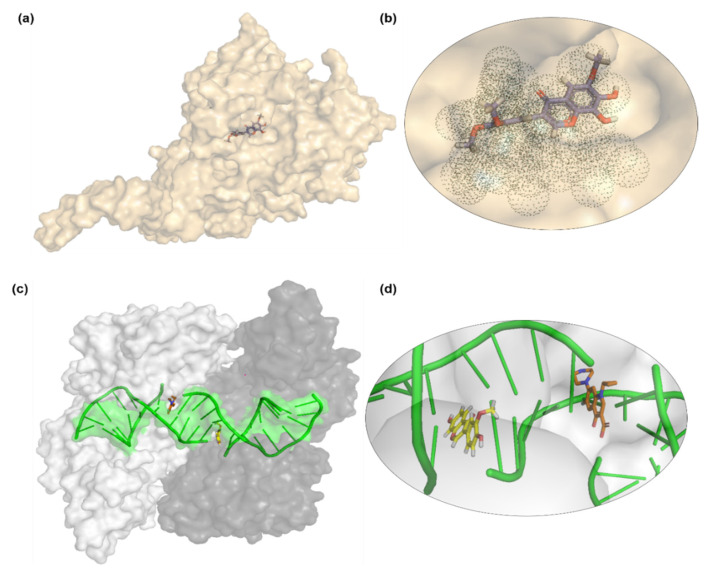
Binding pocket visualization of (**a**,**b**) yeast *S. cerevisiae* lanosterol 14α-demethylase enzyme (a cytochrome P450 enzyme) docked with compound **7** isolated from *Onosma chitralicum*. The enzyme is reported in a co-crystallized form with fluconazole under PDB code 4WMZ. Compound **7** (shown in stick form) is visible in the dotted binding pocket, showing that this compound can act as a potential pro-drug and be modified for fitting in the active site groove. (**c**,**d**) Docked compound **4** is found in the mirror image form of ciprofloxacin, co-crystallized with *S. aureus* topoisomerase-II DNA gyrase (PDB ID: 2XCT), involved in hydrogen bonding to the bound DNA molecule. Ciprofloxacin and compound **4** are shown in sticks, bound DNA in green ribbon, and dimeric enzyme in white and black protomers with surface representation.

The Saccharomyces cerevisiae lanosterol 14α-demethylase (CYP51) (PDB ID: 4WMZ) docked with compound **7** and *S. aureus* topoisomerase-II DNA gyrase (PDB ID: 2XCT) with compound **4** (7,4′-dihydroxy-3′-methoxyisoflavone) with very interesting DNA binding aptitude similar to bound co-crystallized ligand ciprofloxacin, visualized by molecular visualization program PyMOL [30], as shown in Figure 6.

## 3. Materials and Methods

### 3.1. Collection of Plant Material

Chitral city is the source of the *Onosma chitralicum* plant. This plant was recognized by Dr. Nisar Ahmad, Department of Botany, KUST, Kohat, KP, Pakistan, with (PSK 67) as a voucher specimen deposited there.

### 3.2. Extraction, Fractionation, and Bioassays 

The shade and air-dried plant *Onosma chitralicum* (2 kg) were taken and ground to get powder, soaked in methanol (4 L) for 14 days, and were extracted three times during the 14 days at room temperature in the same solvent, and then filtered. The filtrates were evaporated under reduced pressure by vacuum rotary evaporator at 35 °C. The extract was dried and weighed. The crude extract weighed to 80 g, was further suspended in water, and partitioned successively with n-hexane, chloroform, ethyl acetate, and *n*-butanol to obtain their soluble fractions. The weight of each solvent soluble fraction was *n*-hexane (15 g), chloroform (25 g), ethyl acetate soluble (18 g), *n*-butanol (12 g), and aqueous fraction (10 g).

Antibacterial and antifungal activities of the crude extract, other solvent soluble fractions, and the compounds **1**–**7** isolated from *Onosma chitralicum* were investigated. Bacterial strains, such as *Escherichia coli, Pseudomonas aeruginosa*, *Staphylococcus aureus, Salmonella typhi,* and *Bacillus subtilis* were used in antibacterial essay while fungal strains *Aspergillus flavus, Fusarium solani, Aspergillus fumigates, Aspergillus niger,* and *Candida glabrata* were used in antifungal assay. The bacterial and fungal strains used in this study were clinical isolates that were isolated and identified previously at Department of Microbiology of this university. Disc diffusion method was used for antifungal activity, while for antibacterial activities, the agar well diffusion method was used. In the disc diffusion technique, the bacterial culture was streaked on the surface of the solidified agar media in the sterile petri plate. Next, on the sterile disc, 10 μL of the extract or fraction and isolated compounds from their stock solutions were allowed to absorb in independent experiments, and zones of inhibition were measured after 24 h of incubation [31].

In the agar-well diffusion method for bacterial strains, wells of 6 mm were dug in the sterile agar media by using a sterile plastic borer. Next, each well was given a specific number, and bacterial culture was inoculated on the surface of the solidified media. Stock solutions of crude extracts and fractions in one experiment and the isolated compounds in another experiment were added into respective wells. The zones of inhibition were measured after 24 h of incubation at 37 °C in an incubator [32]. Doxycycline and Miconazole were used in the crude assays, whereas, levofloxacin and clotrimazole were used in the isolated compounds screening as standard positive controls, while DMSO was used as a negative control. The zones of inhibition of crude extract, fractions, and the isolated compounds were compared with the zones of inhibition of standard drugs. 

### 3.3. Isolation of Chemical Constituents

The most potent activity was observed in ethyl acetate fraction and was further subjected to separation protocol using column chromatography. The elution through column chromatography was carried out based on the gradient of polarity (*n*-C_6_H_14_ → *n*-C_6_H_14_-EtOAc → pure-EtOAc). As a result, four fractions A, fractions B, fractions C and fractions D were obtained by eluting *n*-C_6_H_14_-EtOAc of different fractions through column chromatography. Fraction A was obtained by using *n*-C_6_H_14_-EtOAc (8:2), consisted of two pure compounds, compound **1** (4′,8-dimethoxy-7-hydroxyisoflavone) [33] and compound **3** (2,3-dihydro-3-hydroxy-7-methoxyflavone) [34] with a ratio of (8.50:1.50 & 7.50:2.50), respectively. Fraction B was also obtained as discussed above with *n*-C_6_H_14_-EtOAc (6.0:4.0), consisted of compound **2** (2,3-dihydro-3,5-dihydroxy-7-methoxyflavone) [35] and compound **4** (7,4′-dihydroxy-3′-methoxyisoflavone) [36], with the ratio of (6.50:3.50 and 6.50:4.0), respectively. Fraction C was eluted by using *n*-C_6_H_14_-EtOAc at the ratio of (9:1) was further subjected to column chromatography. Fraction C afforded only one pure compound, compound **5** (7,4′-dimethoxy-isoflavone) [37]. Fraction D was eluted with *n*- C_6_H_14_-EtOAc by using the ratio of (5:5) and 2 compounds, compound **6** (5,3′,3-trihydroxy-7,4′-dimethoxyflavanone) [38] and compound **7** (5′,7,8-trihydroxy-6,3′,4′-trimethoxyflavanone) [39] with the ratio of (2.50:7.50 & 4.50:5.50) were eluted, respectively.

For isolation of compounds, Eyela (EF-10 model) flash chromatography a type of flash column chromatography technique was used by using silica gel (E. Merck Si 60,230–400 mesh).

### 3.4. Characterizations Techniques

The spectra of UV were reported on spectrophotometric instrument Hitachi UV-3200. IR spectral data were measured using a JASCO 302-A spectrophotometer. EI–MS (*m/z*) were recorded on JEOL JMS-HX-110 mass spectrometer. At 400 MHz, the spectra of ^1^H-NMR were observed on the instrument named Bruker AM-400 AMX spectrometers with the respective frequency data system. TMS of compounds was used as a reference (mainly internally). The spectra of ^13^C-NMR (broadband and DEPT or GASPE) were accomplished on the same instruments at 75 MHz, 100 MHz, and 125 MHz, respectively. The values of the chemical shift were expressed as ppm (δ) and the coupling constants (J) values were expressed in Hz.

### 3.5. Physical and Spectral Data of Isolated Compounds ***1*** to ***7***

*4′,8-dimethoxy-7-hydroxyisoflavone* (**1**): colorless solid. UV λ_max_ (CH_3_Cl) 254,350 nm. IR(KBr) cm^−1^: 1600, 1685, 3450, 1315, ^1^H-NMR (CDCl_3_, 400 MHz) δ: 8.33 (2H s, *J* = 0 Hz), 7.62 (5H d, *J* = 8.3), 6.89 (6H d, *J* = 9.4), 7.47 (2H′ d, *J* = 8.81), 6.94 (3H′ d, *J* = 8.81), 6.94 (5H′ d, *J* = 8.81), 7.47 (6H′ d, *J* = 8.81), 3.81 (8OCH_3_ s, ), 3.79 (4-OCH_3′_s,); ^13^C-NMR (CDCl_3_) δ: 154.2 (C-2), 123.2 (C-3), 181.1(C-4), 123.9 (C-5), 122.5 (C-6), 163.1 (C-7), 145.5 (C-8), 159.6 (C-9), 106.1 (C-10), 123.6 (C-1′), 121 (C-2′), 122 (C-3′), 146.5 (C-4′), 122 (C-5′), 121 (C-6′), 58.4 (8-OCH_3_), 61.3 (4-OCH_3′_); EI-MS *m*/*z* 298. The above physical and spectral data are in complete agreement with the data reported in literature for compound **1 [33]**.

*2,3-dihydro-3,5-dihydroxy-7-methoxyflavone* (**2**): colorless solid. UV λ_max_ (CH_3_Cl) 250,323 nm. IR(KBr) cm^−1^: 1605, 1710, 3250, 1310; ^1^H-NMR (CDCl_3_,400 MHz) δ: 5.14 (2H d, *J* = 11.0 Hz), 4.54 (3H d, *J* = 11.0 Hz), 11.04 (5H s), 6.05 (6H d, *J* = 2.3), 6.18 (8H d, *J* = 2.3 Hz), 7.40 (2H′ m).5, 7.42 (3H′ m), 7.43 (4H′ m) 7.47 (5H′ m), 7.51 (7OCH_3_ s); ^13^C-NMR (CDCl_3_) δ: 77.2 (C-2), 149.7 (C-3), 181.9(C-4), 158.3 (C-5), 121.5 (C-6), 146.3 (C-7), 122.5 (C-8), 156.3 (C-9), 106.9 (C-10), 122.8 (C-1′), 121.9 (C-2′), 123.2 (C-3′), 123.7 (C-4′), 122.2 (C-5′), 121. (C-6′), 58.3 (8OCH_3_); (calcd for C_16_H_12_O_5_284) EI-MS *m*/*z* 284. The above physical and spectral data coincided with the data reported in literature for compound **2 [34]**.

*2,3-dihydro-3-hydroxy-7-methoxyflavone* (**3**): colorless solid. UV λ_max_ (CH_3_Cl) 254,350 nm. IR(KBr) cm^−1^: 1600,1685,3450,1315; ^1^H-NMR (CDCl_3_,400 MHz) δ: 8.33 (2H s, *J*= 0 Hz), 7.62 (5H d, *J* = 8.3), 6.89 (6H d, *J* = 9.4), 7.47 (2H′ d, *J* = 8.81), 6.94 (3H′ d, *J* = 8.81), 6.94 (5H′ d, *J* = 8.81), 7.47 (6H′ d, *J* = 8.81), 3.81 (8OCH_3_ s), 3.79 (4OCH_3_′ s,); ^13^C-NMR (CDCl_3_) δ: 154.2 (C-2), 123.2 (C-3), 181.1(C-4), 123.9 (C-5), 122.5 (C-6), 163.1 (C-7), 145.5 (C-8), 159.6 (C-9), 106.1 (C-10), 123.6 (C-1′), 121 (C-2′), 122 (C-3′), 146.5 (C-4′), 122 (C-5′), 121 (C-6′), 58.4 (8OCH_3_), 61.3 (4OCH_3′_); EI-MS *m*/*z* 268. The above physical and spectral data are in complete agreement with the data reported in literature for compound **3 [35]**.

*7,4′-dihydroxy-3′-methoxyisoflavone* (**4**): colorless solid. UV λ_max_ (CH_3_Cl) 255,336 nm. IR(KBr) cm^−1^: 1610, 1680, 3450, 1325; ^1^H-NMR (CDCl_3_,400 MHz) δ: 5.14 (2H d, *J* = 11.2 Hz), 7.80 (5H d, *J* = 8.9), 6.90 (6H d, *J* = 8.9), 6.49 (8H d, *J* = 2.7 Hz), 7.19 (2H′ d, *J* =1.5), 7.42 (3H′ m), 6.76 (5H′ d, *J* = 8.0) 6.79 (6H′ d, *J* = 8.0), 3.76 (3-OCH_3_); ^13^C-NMR (CDCl_3_) δ: 151.4 (C-2), 153.5 (C-3), 173.5(C-4), 126.5 (C-5), 121.8 (C-6), 165.9 (C-7), 102 (C-8), 156.9 (C-9), 115.8 (C-10), 123.8 (C-1′), 114.3 (C-2′), 148.1 (C-3′), 145.4 (C-4′), 114.1 (C-5′), 118.5 (C-6′); EI-MS *m*/*z* 284. The above physical and spectral data are similar with the data reported in literature for compound **4 [36]**.

*7,4′-dimethoxy-isoflavone* (**5**): colorless solid. UV λ_max_ (CH_3_Cl) 250,323 nm. IR(KBr) cm^−1^: 1605, 1670, 3350, 1340; ^1^H-NMR (CDCl_3_,400 MHz) δ: 7.96 (2H, s), 8.31 (5H d, *J* = 9.3 Hz), 6.97 (6H dd, *J* = 9.3,2.5), 6.87 (8H d, *J* = 2.5), 7.40 (2H′ d, *J* = 8.1), 7.42 (5H′ d, *J* = 8.4), 7.40 (6H′ d, *J* = 8.1), 3.85 (7OCH_3_ s), 3.89 (4OCH_3_′ s), ^13^C-NMR (CDCl_3_) δ: 150.2 (C-2), 123.1 (C-3), 181.3 (C-4), 123.8 (C-5), 122.8 (C-6), 131.8 (C-7), 122.5 (C-8), 159.6 (C-9), 106.1 (C-10), 123.6 (C-1′), 121 (C-2′), 122 (C-3′), 131.1 (C-4′), 122 (C-5′), 121 (C-6′), 61.2 (4OCH_3_′) 61.3 (8-OCH_3_); EI-MS *m*/*z* 282. The above physical and spectral data are in complete agreement with the data reported in literature for compound **5** [37].

*5,3′,3-trihydroxy-7,4′-dimethoxyflavanone* (**6**): colorless solid. UV λ_max_ (CH_3_Cl) 260,388 nm. IR(KBr) cm^−1^: 1605, 1680, 3410, 1340; ^1^H-NMR (CDCl_3_,400 MHz) δ: 4.59 (2H d, *J* = 11.5 Hz), 5.04 (3H d, *J* = 11.5 Hz), 11.24 (5H s) 6.19 (6H d, *J* = 2.8), 6.18 (8H d, *J* = 2.8), 6.96 (2H′ d, *J* = 2.8), 6.91 (5H′ d, *J* = 7.8), 7.09 (6H′ d,d *J* = 7.8,2.8), 3.81 (7OCH_3_ s,), 3.89 (4OCH_3_′ s); ^13^C-NMR (CDCl_3_) δ: 85.6 (C-2), 77.4 (C-3), 194.9(C-4), 166.6 (C-5), 99.1(C-6), 167.8 (C-7), 98.7 (C-8), 168.9 (C-9), 100.2 (C-10), 128.8 (C-1′), 109.1 (C-2′), 144.5 (C-3′), 145.6 (C-4′), 115.6 (C-5′), 122.1 (C-6′), 56.8 (4OCH_3_^’^) 56.9 (8-OCH_3_); EI-MS *m*/*z* 360. The above physical and spectral data coincided with the data reported in literature for compound **6 [38]**.

*5′,7,8-trihydroxy-6,3′,4′-trimethoxyflavanone* (**7**): colorless solid. UV λ_max_ (CH_3_Cl) 250,330 nm. IR(KBr) cm^−1^: 1603, 1695, 3310, 1350; ^1^H-NMR (CDCl_3_,400 MHz) δ: 8.35(2H s, 6.71 (5H s), 3.89 (3OCH_3_′ s), 3.78 (4OCH_3_′ s), 7.47 (6H′ d, *J* = 2.9), 3.47 (6OCH_3_ s,); ^13^C-NMR (CDCl_3_) δ: 156.3 (C-2), 123.8 (C-3), 185.6(C-4), 118.1 (C-5), 154.9 (C-6), 151.1 (C-7), 136.6 (C-8), 159.8 (C-9), 107.1 (C-10), 124.8 (C-1′), 124.4 (C-2′), 149.1 (C-3′), 149.9 (C-4′), 136.8 (C-5′), 136.4 (C-6′), 60.9(3OCH_3_′), 59.1 (4-OCH_3_′) 58.1 (6-OCH_3_); EI-MS *m*/*z* 332. The above physical and spectral data are similar with the data reported in literature for compound **7 [39]**.

### 3.6. Docking Analysis Protocol

The molecular docking studies of all seven compounds (compounds **1**–**7**) were carried out to investigate the putative binding interaction within the target proteins. The starting three-dimensional (3D) structure of *S. aureus* tyrosyl-tRNA synthetase (PDB id: 1JIJ) and topoisomerase II DNA gyrase (PDB id: 2XCT) and beta-hydroxyacyl-ACP dehydratase HadAB (PDB id: 4RLT) were obtained from the Protein Data Bank (PDB) [40]. Ligand molecules were sketched using the Chem Draw Professional v17 (PerkinElmer, Akron, OH, USA). The molecules were converted into 3D using Chem3D (PerkinElmer, Akron, OH, USA) [41]. Before performing the docking protocol, chemically correct models of the ligands were generated using the OMEGA module of OpenEye Scientific Software, and the receptor structures were prepared using the MAKERECEPTOR Wizard Module of Openeye Scientific Software (Santa Fe, NM, USA) [42]. Molecular docking was carried out using the FRED ligand-docking module. FRED requires a set of input conformers for each compound. The conformers of each ligand were generated by OMEGA 3.0.0 (Santa Fe, NM, USA) [43]. Default settings of OMEGA were used for the generation of multi-conformers. Receptor grids were generated using the PDB2Receptor grid generation module. Grids were generated for the prepared proteins. For the *S. aureus* gyrase complex, the grid was generated around ciprofloxacin, while for the *S. aureus* tyrosyl-tRNA synthetase complex, the grid was generated around co-crystal SB-239629 ligand. Moreover, in the case of beta-hydroxyacyl-ACP dehydratase HadAB grid box was selected around Fisetin. In the case of *S. cerevisiae* co-crystallized, the grid was generated around co-crystal fluconazole ligand. The boundary box was set at default value, which was spacious enough to encompass the binding regions in target proteins. The docking protocol was optimized using the re-docking of the co-crystal ligand within the active site of the target protein. FRED generated ten poses for each ligand and the pose with the lowest Chemguass4 was selected for further analysis. Binding interactions of best-docked poses were visualized using Discovery Studio client v16.1.0 [44].

## 4. Conclusions

The present work concluded the bioassay’s guided phytochemical investigations of *Onosma chitralicum*, the most active fraction against different microbes was the ethyl acetate fraction with inhibition zone of 16.0 ± 0.47 mm against *S. typhi*. The fraction was also active against fungal strains *A. flavus* and *F. solani* with inhibition zones of 60 ± 0.94 mm and 57 ± 0.51 mm, respectively. The isolation from ethyl acetate soluble fraction resulted in seven compounds, **1** to **7,** as potential pharmaceutical targets. Compounds **1** (4′, 8-dimethoxy-7-hydroxyisoflavone), **6** (5,3′,3-trihydroxy-7,4′-dimethoxyflavanone), and **7** (5′, 7,8-trihydroxy-6,3′,4′-trimethoxyflavanone) were found to be more active against *S. aureus* and *S. typhi*. Compound **1** inhibit *S. typhi* and *S. aureus* to 10 ± 0.21 mm and 10 ± 0.45 mm, whereas compound **6** showed inhibition to 10 ± 0.77 mm and 9 ± 0.20 mm, respectively, and compound **7** inhibit *S. aureus* to 6 ± 0.36 mm. Docking studies also support the antibacterial activity by binding to bacterial enzymes beta-hydroxyacyl dehydratase (HadAB complex) and tyrosyl-tRNA synthetase. Compound 7 also showed best docking results to the antifungal fluconazole binding enzyme Lanosterol 14α-demethylase. These findings lead to the identification of chemical scaffolds, which can lead to new broad-spectrum antimicrobial drugs targeted against functionally important proteins of human pathogens, and modification to existing inhibitors of these important proteins to improve affinity and potency. However, a further study (in vivo) would help in supporting further insight into the pharmacological properties of these isolated compounds. 

## 5. Future Perspectives

Molecular docking studies give us putative information about the target of interest. Shading light on the antibacterial and antifungal activities, we performed molecular docking to gain insight into the putative binding interaction with possible target protein. In future studies, we will aim to screen these selected compounds against the best-identified target protein.

## Figures and Tables

**Table 1:** Antibacterial activities showing zone of inhibition (mm) ± standard error mean of various fractions (15 mg/mL) of *Onosma chitralicum.*

S.#	Fractions	*E. coli*	*P. aeruginosa*	*S.* *aureus*	*S. typhi*	*B. subtilis*
1.	Crude	13.0 ± 0.46	8.0 ± 0.23	13.0 ± 0.44	17.0 ± 0.52	12.0 ± 0.39
2.	*n*-hexane (C_6_H_14_)	11.0 ± 0.61	2.0 ± 0.30	8.0 ± 0.32	7.0 ± 0.32	10.0 ± 0.67
3.	Chloroform (CHCl_3_)	4.0 ± 0.42	6.0 ± 0.40	6.0 ± 0.46	3.0 ± 0.22	2.0 ± 0.25
4.	Ethyl acetate (EtoAc)	12.0 ± 0.32	3.0 ± 0.33	12.0 ± 0.21	16.0 ± 0.47	11.0 ± 0.41
5.	*n*-butanol (BuOH)	3.0 ± 0.22	4.0 ± 0.32	5.0 ± 0.53	3.0 ± 0.14	2.0 ± 0.12
6.	Aqueous	3.0 ± 0.23	1.0 ± 0.15	3.0 ± 0.26	5.0 ± 0.28	2.0 ± 0.16
7.	Doxycycline (standard)	16.0 ± 0.12	10.9 ± 0.15	20.0 ± 0.26	24.0 ± 0.13	22.0 ± 0.29

**Table 2:** Antifungal activities showing zone of inhibition (mm) ± standard error mean of various fractions (15 mg/mL) of *Onosma chitralicum.*

S.#	Fractions	*A. flavus*	*F. Solani*	Aspergillus *fumigatus*	*A. niger*	*Candida glabrata*
1.	Crude	65 ± 0.56	63 ± 0.78	45 ± 0.83	58 ± 0.72	40 ± 0.54
2.	*n*-hexane (C_6_H_14_)	50 ± 0.32	40 ± 0.86	26 ± 0.52	54 ± 0.28	36 ± 0.91
3.	Chloroform (CHCl_3_)	31 ± 0.67	29 ± 0.54	30 ± 0.40	23 ± 0.35	31 ± 0.22
4.	Ethyl acetate (EtoAc)	60 ± 0.94	57 ± 0.51	40 ± 0.88	48 ± 0.30	33 ± 0.82
5.	*n*-butanol (BuOH)	37 ± 0.59	24 ± 0.29	21 ± 0.47	35 ± 0.66	22 ± 0.65
6.	Aqueous	21 ± 0.29	10 ± 0.30	21 ± 0.25	17 ± 0.56	20 ± 0.43
7.	Miconazole (standard)	100	100	100	100	100

**Table 3:** Antibacterial activities showing zone of inhibition (mm) ± standard error mean of compounds **1**–**7** (28 μg/mL) isolated from *Onosma chitralicum.*

S.#	Compounds	*E. coli*	*P. aeruginosa*	*S.* *aureus*	*S. typhi*	*B. subtilis*
**1.**	Compd. **1**	7 ± 0.23	2 ± 0.42	10 ± 0.45	10 ± 0.21	2 ± 0.66
**2.**	Compd. **2**	8 ± 0.57	0	5 ± 0.45	0	0
**3.**	Compd. **3**	0	2 ± 0.20	4 ± 0.76	0	7 ± 0.25
**4.**	Compd. **4**	9 ± 0.23	1 ± 0.87	4 ± 0.61	5 ± 0.19	5 ± 0.32
**5.**	Compd. **5**	3 ± 0.54	2 ± 0.46	3 ± 0.21	1 ± 0.23	6 ± 0.32
**6.**	Compd. **6**	6 ± 0.88	2 ± 0.65	9 ± 0.20	10 ± 0.77	3 ± 0.51
**7.**	Compd. **7**	6 ± 0.37	0	6 ± 0.36	2 ± 0.29	0
**8.**	Levofloxacin	17 ± 0.17	10 ± 0.15	23 ± 0.34	21 ± 0.20	19 ± 0.13

**Table 4:** Antifungal activities of compounds **1**–**7** (28 μg/mL) isolated from *Onosma chitralicum.*

S. #	Compounds	*A. flavus*	*F. Solani*	*A. fumigatus*	*A. niger*	*C. glabrata*
**1.**	Compd. **1**	+,+	+,+	+,+	+,+	+,+
**2.**	Compd. **2**	+,+	−,−	−,−	−,−	−,−
**3.**	Compd. **3**	−,−	−,−	−,−	−,−	−,−
**4.**	Compd. **4**	+,+	+,+	−,−	+,+	−,−
**5.**	Compd. **5**	+,+	+,+	−,−	+,+	−,−
**6.**	Compd. **6**	+,+	+,+	−,−	+,+	−,−
**7.**	Compd. **7**	+,+	−,−	−,−	+,+	−,−
**8.**	Clotrimazole	+,+,+,+	+,+,+,+	+,+,+,+	+,+,+,+	+,+,+,+

Key: (−,−) 100% growing; (+,+) 25% growing; (+,+,+,+) not growing.

## Data Availability

The data presented in this study are available on request from the corresponding author.

## Sample Availability

Samples of the compounds are available and can be obtained from authors S.A.K. and I.A.
